# Ethics and open access

**DOI:** 10.1002/nop2.21

**Published:** 2015-07-14

**Authors:** Roger Watson

Open access publishing has advantages for authors. They can share their work freely without infringing copyright restrictions and thereby, reach a wider audience and increase the potential of their work to be read and cited. The advantages of open access publishing are clear to university and research funding bodies and an increasing number of countries and organisations are making moves to encourage and oblige open access to research as explained in my last editorial (Watson 2015). This is to increase the value of published work to the general public who, through taxation and charitable donations, indirectly fund the work.

I am very much in favour of open access publication. However, it is not appreciated by many academics that there is a cost attached to publishing scientific articles. Many are insensible to the cost as they have free access to all the important published content in their field through their university libraries. On the occasions that their university does not hold a subscription, and they are faced with the cost of downloading an article, they see what publishers charge to view and download an article. Many are surprised at the cost and cannot understand why they have to pay as the article is already published. They do not understand that the extent to which something is published – and available to them – means that a publisher has already borne the cost which includes editorial work, production, website maintenance and the salaries of those who have to provide these functions. If the article is available to them via their university library then their university will have borne the cost of purchasing a subscription.

Concomitantly, and traditionally, publishers hold the copyright to published articles. This comes as another shock to many academics, who have done the work that led to their articles, including writing them, and who fail to see why they cannot do what they like with their published work. There is an internal logic to this argument which does not stand up to the test of external factors such as those described above. The publisher has borne the cost of producing and making available articles; without maintaining some control over the use of the published article, how do they recoup their costs? Some complain of the profits publishers make – invariably described as 'excessive' – without seeing the operation as a whole and the fact that in the UK, for example, the publishing industry is a major employer and contributor to the economy. The same people who complain about these job‐sustaining profits are often the same who take to the picket lines when their own jobs are under threat; they compound their ignorance of the business model of the publishing industry with their ignorance of the fact that university education also costs money. Expecting free access to published articles is analogous to expecting free access to the postal service simply because you wrote the letter that you wish to post.

## The open access movement

Enter the open access movement. It is hard to specify its beginnings but impossible to ignore its influence. Open access to published scientific outputs is now considered 'the norm' and, as described in my previous editorial (Watson 2015), the open access movement has swung political opinion in its favour such that research councils and higher education funding bodies now insist that publications emanating from research they fund and which is assessed by them is published open access. Notwithstanding the green route to open access, there is a supreme irony in the fact that, to publish open access by the gold route (i.e. freely available to read and distribute immediately and in its final published form) in prestigious journals costs more than the average academic can afford. In addition, some publishers have been able to 'double dip' by charging some universities to access content which is subsequently made available open access, although publishers are taking steps to address this (see Wiley's policy regarding subscription pricing for hybrid journals, http://onlinelibrary.wiley.com/journal/10.1002/%28ISSN%292054-1058/homepage/article_publication_charges.htm). The costs are no longer insensible, and in the UK universities are spending vast amounts of money supporting open access either by subscription, paying for the publication of articles or, in the case of the green route, setting up expensive repositories and dedicating staff time to ensuring the open access policies are adhered to in order that staff publications meet the specifications of funding bodies. I have no figures to support my argument but I feel safe from contradiction in saying that the overall costs of publishing scientific articles has increased several fold. Did the open access movement anticipate this? I doubt it.

## Ethics

The sharp practices of some open access publishers were covered in my previous editorial (Watson 2015) and some of these are, undoubtedly, fraudulent activities. At another level many of these activities breach publication ethics as, essentially, individuals are paying to publish their article, as distinct from paying to publish their article open access. In some cases editorial and peer‐review scrutiny are non‐existent or very ‘light touch’. As described previously, this is an enormous ‘industry’ taking academics’ money from them and misleading the public who may be misled into thinking that these non‐peer‐reviewed articles are equal in status to peer‐reviewed articles. Another direct, if unintended, consequence of the open access movement.

Publishers and editors involved in open access publishing need to ensure, and let it be known they ensure, that they separate the editorial processes in journals offering open access from the pay to publish open access processes. While the Committee on Publication Ethics have no guidance specific to editorial processes for open access, they do have relevant codes of conduct and the one for publishers (2011a) refers to ‘transparency and integrity’ and the same theme can be seen in their code for editors (2011b) which says that editors must ‘preclude busin‐ess needs from compromising intellectual and ethical standards’. If editors bowed to publishers' pressures to publish specific articles or more articles for business reasons then a breach of publication ethics would occur. At *Nursing Open*, and across the Wiley stable, we specify that we do keep these processes separate. In our author guidelines for *Nursing Open*, we state: ‘After review and acceptance, you will be prompted to sign the Open Access Agreement form’ and for example, in the *JAN* author guidelines it says:

The Editorial Office should not be informed of the decision to publish Online Open until the manuscript has been accepted. All papers go through the journal's standard peer‐review process and are accepted or rejected based on their own merit.

At *Nursing Open* we are determined to ensure that our open access processes do not compromise an ethical approach to publishing and I reiterate from my first editorial (Watson 2014) our commitment to integrity which I described as:

*Integrity* – *Nursing Open* will operate according to the highest standards of authorship, peer review, editing and publishing. The ‘pay to publish’ aspect of the journal will be entirely separate from judging the scientific worth of articles. We provide a clear standard template for submission to which all articles must conform, and all submissions will be peer reviewed and edited. The process of publication and any problems or disputes arising throughout that process and after publication will be handled with fairness and equity according to the COPE guidelines.
